# Serotype Distribution and Antimicrobial Resistance of Streptococcus agalactiae Isolates in Nonpregnant Adults with Streptococcal Toxic Shock Syndrome in Japan in 2014 to 2021

**DOI:** 10.1128/spectrum.04987-22

**Published:** 2023-02-14

**Authors:** Tadayoshi Ikebe, Rumi Okuno, Yumi Uchitani, Mami Takano, Takahiro Yamaguchi, Hitoshi Otsuka, Yu Kazawa, Shohei Fujita, Ayaka Kobayashi, Yoshimi Date, Junko Isobe, Emi Maenishi, Makoto Ohnishi, Yukihiro Akeda, the Working Group for Beta-Hemolytic Streptococci in Japan

**Affiliations:** a National Institute of Infectious Diseases, Tokyo, Japan; b Tokyo Metropolitan Institute of Public Health, Tokyo, Japan; c Oita Prefectural Institute of Health and Environment, Oita, Japan; d Osaka Institute of Public Health, Osaka, Japan; e Yamaguchi Prefectural Institute of Public Health and Environment, Yamaguchi, Japan; f Fukushima Prefectural Institute of Public Health, Fukushima, Japan; g Kanagawa Prefectural Institute of Public Health, Kanagawa, Japan; h Toyama Institute of Health, Toyama, Japan; Hokkaido Institute of Public Health; Hokkaido Institute of Public Health; Akita Prefectural Research Center for Public Health and Environment; Iwate Prefectural Research Institute for Environmental Sciences and Public Health; Iwate Prefectural Research Institute for Environmental Sciences and Public Health; Yamagata Prefectural Institute of Public Health, Miyagi Prefectural Institute of Public Health and Environment, Sendai City Institute of Public Health; Niigata Prefectural Institute of Public Health and Environmental Sciences; Niigata City Institute of Public Health and Environment; Niigata City Institute of Public Health and Environment; Ibaraki Prefectural Institute of Public Health; Gunma Prefectural Institute of Public Health and Environmental Sciences; Gunma Prefectural Institute of Public Health and Environmental Sciences; Nagano Environmental Conservation Research Institute), Saitama Prefectural Institute of Public Health; Kawasaki City Institute for Public Health; Yokohama City Institute of Public Health; Ciba City Institute of Environmental Sciences and Public Health; Saitama City Institute of Health Science and Research; Shizuoka City Institute of Environmental Sciences and Public Health; Shizuoka City Institute of Environmental Sciences and Public Health; Tochigi Prefectural Institute of Public Health and Environmental Science; Hamamatsu City Health and Environment Research Institute; Chiba Prefectural Institute of Public Health; Yokosuka Institute of Public Health; Utsunomiya City Institute of Public Health and Environment; Mie Prefecture Health and Environment Research Institute; Mie Prefecture Health and Environment Research Institute; Aichi Prefectural Institute of Public Health; Toyota City Public Health Center; Gifu Prefectural Research Institute for Health and Environmental Sciences; Gifu Prefectural Research Institute for Health and Environmental Sciences; Ishikawa Prefectural Institute of Public Health and Environmental Science; Ishikawa Prefectural Institute of Public Health and Environmental Science; Toyama City Public Health Center; Hyogo Prefectural Institute of Public Health Science; Kyoto City Institute of Health and Environmental Science; Kobe City Institute of Health Science; Wakayama City Institute of Public Health; Wakayama Prefectural Research Center of Environment and Public Health; Shiga Prefectural Institute of Public Health; Shiga Prefectural Institute of Public Health; Amagasaki City Institute of Public Health; Amagasaki City Institute of Public Health; Tottori Prefectural Institute of Public Health and Environment; Shimane Prefectural Institute of Public Health and Environmental Science; Okayama Prefectural Institute for Environmental Science and Public Health; Hiroshima Prefectural Technology Research Institute, Public Health and Environment Center; Hiroshima Prefectural Technology Research Institute, Public Health and Environment Center, Hiroshima City Institute of Public Health, Kagawa Prefectural Research Institute for Environmental Science and Public Health; Tokushima Prefectural Public Health Pharmaceutical and Environmental Sciences Center; Tokushima Prefectural Public Health Pharmaceutical and Environmental Sciences Center; Ehime Prefectural Institute of Public Health and Environmental Science; Kochi Prefectural Public Health and Environmental Science Research Institute; Kochi Prefectural Public Health and Environmental Science Research Institute; Nagasaki Prefectural Institute of Environment and Public Health; Saga Prefectural Institute of Public Health and Pharmaceutical Research; Kumamoto Prefectural Institute of Public Health and Environmental Science; Kitakyushu City Institute of Health and Environmental Sciences; Okinawa Prefectural Institute of Health and Environment; Okinawa Prefectural Institute of Health and Environment; Nevada State Public Health Laboratory

**Keywords:** *Streptococcus agalactiae*, streptococcal toxic shock syndrome, invasive infections, serotype, antimicrobial resistance, vaccine

## Abstract

The incidence of streptococcal toxic shock syndrome (STSS) due to group B Streptococcus (GBS) has been increasing annually in Japan and is becoming a serious challenge. Furthermore, in recent years, penicillin- or clindamycin-resistant strains used in treating streptococcal toxic shock syndrome have been reported. However, no report analyzed >100 isolates of group B Streptococcus causing streptococcal toxic shock syndrome. Therefore, we aimed to perform serotyping and antimicrobial susceptibility testing of 268 isolated group B Streptococcus strains from streptococcal toxic shock syndrome cases involving nonpregnant adult patients in Japan between 2014 and 2021. The most prevalent serotype was Ib, followed by serotypes V, III, and Ia. Seven isolates were resistant to penicillin G, and 17.9% (48 isolates) were resistant to clindamycin. Of the penicillin-resistant group B Streptococcus isolates, 71.4% (5 isolates) were clindamycin resistant. In addition, group B Streptococcus strains resistant to penicillin and clindamycin were isolated from patients with streptococcal toxic shock syndrome. Therefore, before these strains become prevalent, introduction of the group B Streptococcus vaccine is essential for disease prevention.

**IMPORTANCE** Group B Streptococcus (GBS) has been increasingly associated with invasive disease in nonpregnant adults. Such infections are responsible for substantial morbidity and mortality, particularly in individuals with underlying chronic conditions. Streptococcal toxic shock syndrome (STSS) is a severe invasive infection characterized by the sudden onset of shock, multiorgan failure, and high mortality. In this study, we assessed 268 GBS-related STSS cases in nonpregnant adults in Japan between 2014 and 2021. Serotype Ib was the most prevalent, followed by serotypes V, III, and Ia, which were identified in more than 80% of STSS isolates. We found that 48 clindamycin-resistant strains and 7 penicillin G-resistant strains were isolated between 2014 and 2021. We believe that our study makes a significant contribution to the literature because we show that the GBS vaccine, particularly the hexavalent conjugate vaccine, is important to reduce the number of patients with STSS.

## INTRODUCTION

Streptococcus agalactiae (group B Streptococcus [GBS]) is a Gram-positive bacterium classified in Lancefield group B. GBS is found in the gastrointestinal tracts and vaginas of asymptomatic humans. It is well established as a colonizing agent in pregnant women. It is an important cause of neonatal sepsis and meningitis (1). Nevertheless, in the past few decades, GBS has been increasingly associated with invasive diseases in nonpregnant adults (2–5). Such infections are responsible for substantial morbidity and mortality, particularly in individuals with underlying chronic conditions (5).

Streptococcal toxic shock syndrome (STSS) is a severe invasive infection characterized by the sudden onset of shock, multiorgan failure, and high mortality (6, 7). It is caused by group A Streptococcus (Streptococcus pyogenes) (8, 9). GBS causes invasive streptococcal infections, including STSS, similar to group A Streptococcus (6, 10). Furthermore, there are significant differences between GBS-related STSS and invasive GBS (iGBS), especially in prognosis in Japan (7, 11). Serotype is a factor associated with invasiveness and prognosis often reported in adult iGBS but limited in GBS STSS (1, 12). An approach to treating severe invasive infections, such as STSS, is combining penicillin and clindamycin (13). However, clinical GBS isolates with decreased susceptibility to penicillin have been reported in several countries (7, 14–17). Resistance to clindamycin varies widely: up to 74.1% in China (18) and 65.9% in Taiwan (19).

Based on structural differences in the polysaccharide capsule, GBS isolates can be divided into 10 serotypes: Ia, Ib, and II to IX (20). GBS-related STSS is a devastating illness with a high mortality rate. However, as this infection is rare, no study has analyzed >100 isolates of GBS causing STSS, and the serotype prevalent in STSS isolates remains unknown. Herein, we aimed to describe the bacteriological analysis of 268 GBS-related STSS cases in nonpregnant adults.

## RESULTS

### Isolates.

The total number of STSS cases in 2014 to 2021 was 543. Of these, we collected 268 strains (by year: 2014, 16; 2015, 16; 2016, 26; 2017, 46; 2018, 42; 2019, 52; 2020, 40; 2021, 30) (Table 1), accounting for 49% of the total, isolated from nonpregnant adult patients with GBS-related STSS between 2014 and 2021. Patients were 28 to 99 years of age (interquartile range, 61 to 84 years); their average age was 71.6 years, and the median age was 73 years. Additionally, 53.7% of patients (144/268) in the study were males. The 7-day case fatality rate was 24.6% (66/268). Fatality rates by age group revealed higher fatality in the older age groups (18 to 50 years old, 13.0% [3/23]; 51 to 60 years old, 13.6% [6/44]; 61 to 70 years old, 24.6% [14/57]; 71 to 80 years old, 24.5% [12/49]; 81 to 90 years old, 31.2% [24/77]; ≥91 years old, 38.9% [7/18]). All patients experienced clinical shock, and some had renal impairment (190 cases [70.9%]), disseminated intravascular coagulation (163 cases [60.8%]), liver involvement (92 cases [34.3%]), acute respiratory distress syndrome (52 cases [19.4%]), generalized erythematous macular rash (8 [3.0%]), or soft tissue necrosis (95 [35.4%]) (Table 1). GBS was isolated from 256 (95.5%) blood samples, 11 (4.1%) sterile aspirate samples from different depths of bodies and internal body sites, and 1 (0.4%) joint fluid sample collected from 268 patients.

**TABLE 1:** Demographics of the cases

Demographic	Result for cases
Isolates, no. by yr(s)	
2014–2021	268
2014	16
2015	16
2016	26
2017	46
2018	42
2019	52
2020	40
2021	30
Age, yr	28–99
Avg	71.6
Median	73
Interquartile range	61–84
Sex, no. (%)	
Male	144 (53.7)
Female	124 (46.3)
7-day mortality, no. (%)	66 (24.6)
Clinical manifestations, no. (%)	
Renal impairment	190 (70.9)
Disseminated intravascular coagulation	163 (60.8)
Liver involvement	92 (34.3)
Acute respiratory distress syndrome	52 (19.4)
Generalized erythematous macular rash	8 (3.0)
Soft tissue necrosis	95 (35.4)

**FIG 1:**
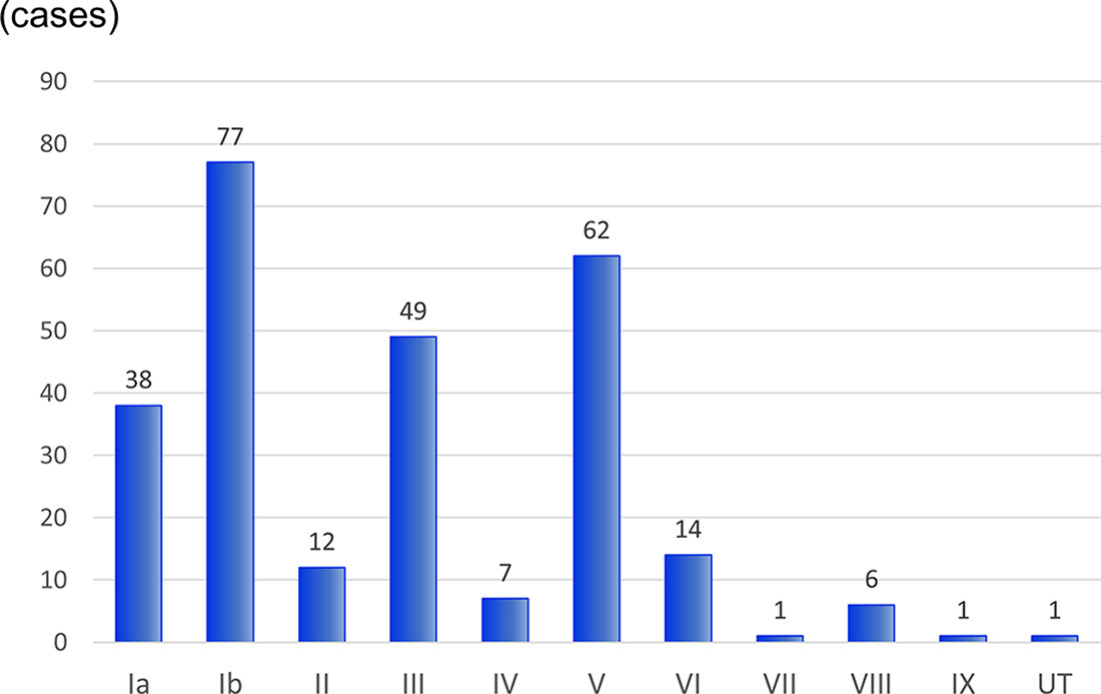
Serotype distribution in 268 GBS isolates from patients with STSS between 2014 and 2021. UT, untypeable.

We determined the serotypes of the 268 STSS isolates by performing agglutination tests using antisera against each capsular type (Fig. 1). The most prevalent serotype was Ib (28.7% [77/268]), followed by serotypes V (23.1% [62/268]), III (18.3% [49/268]), and Ia (14.2% [38/268]), which were identified in over 80% of STSS isolates. No significant temporal changes in serotypes were observed during this period. Therefore, there is no association between death prognosis and the serotype.

### Antimicrobial susceptibility test.

We measured the antimicrobial susceptibility of 268 isolates to 11 drugs (Table 2). Seven isolates (7/268 [2.6%]) were resistant to penicillin G. The highest number of isolates was resistant to ciprofloxacin (90/268 [33.6%]), followed by erythromycin (81/268 [30.2%]), clindamycin (48/268 [17.9%]), penicillin G (7/268 [2.6%]), cefazolin (5/268 [1.9%]), ampicillin (4/268 [1.5%]), and cefotaxime (1/268 [0.4%]). All 268 GBS isolates were susceptible to the following agents (with MIC ranges shown in parentheses): meropenem (0.015 to 0.25 μg/mL), vancomycin (0.25 to 1 μg/mL), daptomycin (≤0.06 to 1 μg/mL), and linezolid (1 to 2 μg/mL).

**TABLE 2:** MIC_50_ and MIC_90_ values and percentage of antimicrobial resistance for Streptococcus agalactiae clinical isolates (*n* = 268)

Antimicrobial agent	Resistance breakpoint (μg/mL)^*a*^	% of resistance (no. of isolates)	MIC (μg/mL)		
			Range	MIC_50_	MIC_90_
Penicillin G	≥0.25	2.6 (7)	0.015 to 0.5	0.03	0.06
Ampicillin	≥0.5	1.5 (4)	0.06 to 0.5	0.12	0.12
Cefazolin	≥1	1.9 (5)	0.06 to 2	0.12	0.25
Cefotaxime	≥1	0.4 (1)	0.03 to 1	0.06	0.12
Clindamycin	≥1	17.9 (48)	0.12 to ≥16	0.12	≥16
Erythromycin	≥1	30.2 (81)	0.12 to ≥16	0.12	≥16
Ciprofloxacin	≥8	33.6 (90)	0.5 to ≥32	1	≥32
Meropenem	≥1	0 (0)	0.015 to 0.25	0.03	0.06
Vancomycin	≥2	0 (0)	0.25 to 1	0.5	1
Daptomycin	≥2	0 (0)	≤0.06 to 1	0.25	0.5
Linezolid	≥4	0 (0)	1 to 2	2	2

a Breakpoints for antimicrobial resistance were determined according to the Clinical and Laboratory Standards Institute M100-S23 guidelines (26).

### Erythromycin- and clindamycin-resistant isolates.

Of 268 isolates, 83 (31.0%) were resistant to erythromycin or clindamycin. Forty-six isolates (17.2%) were resistant to erythromycin and clindamycin. Two isolates (0.7%) were resistant to clindamycin and susceptible to erythromycin. Thirty-five isolates (13.1%) were resistant to erythromycin and susceptible to clindamycin, of which 11 were erythromycin induced (Table 3). No association was observed between death prognosis and drug resistance.

**TABLE 3:** Characteristic of erythromycin- and clindamycin-resistant isolates

Isolate type(s)^*a*^	No. (%) of isolates
Ery^r^ or Cli^r^	83 (31.0)
Ery^r^ Cli^r^	46 (17.2)
Cli^r^ Ery^s^	2 (0.7)
Ery^r^ Cli^s^	35 (13.1)
Ery-inducible Cli^r^	11 (4.1)
Not Ery inducible	24 (9.0)
Ery^s^ Cli^s^	185 (69.0)
Ery^r^	81 (30.2)
Serotype	
III	37
Ib	13
Ia	12
V	12
II	5
VI	1
VIII	1
Ery^r^ gene(s)	
*mefA*/*E*	17
*ermA*	12
*ermB*	43
*mefA* and *msrD*	7
*ermA* and *msrD*	2
Cli^r^	48 (17.9)
Serotype	
III	18
Ib	11
V	9
Ia	6
VI	2
II	1
VIII	1
Cli^r^ gene(s)	
* linB*	2
* ermA*	1
* ermB*	43
* ermA* and *msrD*	2
Phenotype carrying each resistance gene	
*ermB*: Ery^r^ Cli^r^	43
*linB*: Cli^r^ Ery^s^	2
*ermA*	
Ery^r^ Cli^r^	1
Ery-inducible Cli^r^	11
*mefA*/*E*: Ery^r^ Cli^s^	17
*mefA*/*E* and *msrD*: Ery^r^ Cli^s^	7
*ermA* and *msrD*: Ery^r^ Cli^r^	2

a Ery^r^ and Ery^s^, erythromycin resistant and susceptible, respectively; Cli^r^ and Cli^s^, clindamycin resistant and susceptible, respectively.

**FIG 2:**
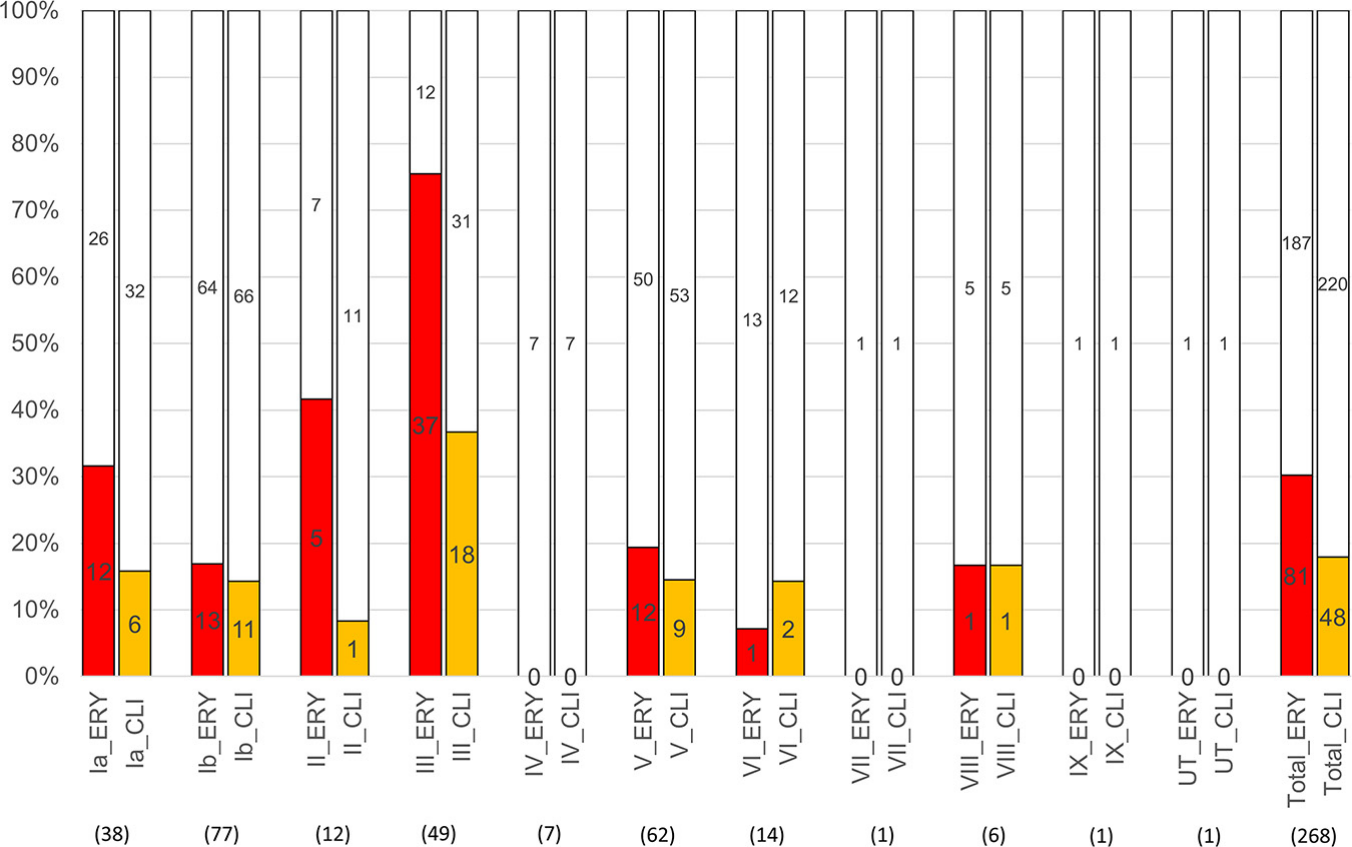
Proportions of erythromycin- and clindamycin-resistant isolates in each serotype. The proportion of each serotype of erythromycin and clindamycin resistance in 268 strains isolated from nonpregnant adult patients with GBS-related STSS. Numbers in the boxes represent the number of isolates. ERY, erythromycin; CLI, clindamycin. The number of isolates is presented in parentheses. The red and orange bars indicate the proportion of erythromycin- and clindamycin-resistant isolates in each serotype, respectively.

Overall, 81 erythromycin-resistant strains (30.2%) were isolated between 2014 and 2021 (Table 3). Of the 81 erythromycin-resistant isolates, 37 were serotype III, 13 were b, 12 were Ia, 12 were V, 5 were II, 1 was VI, and 1 was VIII (Table 2 and Fig. 2). Surprisingly, 75.5% of the serotype III isolates were resistant to erythromycin (Fig. 2). The rate of erythromycin resistance in serotype III STSS isolates was higher than those in other serotype isolates (*P* < 0.001) (Fig. 2). Of the 81 erythromycin-resistant isolates, 24, 9,14, and 43 carried the *mefA*/*E*, *msrD*, *ermA*, and *ermB* genes, respectively (Table 3). The strains carrying the *msrD* gene also carry *mefA*/*E* (seven strains) or the *ermA* gene (two strains).

Overall, 48 clindamycin-resistant strains (17.9%) were isolated between 2014 and 2021 (Table 3). Of the 48 clindamycin-resistant isolates, 18, 11, 9, 6, 2, 1, and 1 were serotypes III, Ib, V, Ia, VI, II, and VIII, respectively (Table 3 and Fig. 2). The rate of clindamycin resistance in serotype III STSS isolates was higher than in other serotype isolates (*P* < 0.001) (Fig. 2). Of the 48 clindamycin-resistant isolates, 2, 2, 3, and 43 carried the *linB*, *msrD*, *ermA*, and *ermB* genes, respectively (Table 3). Two isolates of three strains carrying *ermA*, which are resistant to clindamycin, had *msrD*.

All 43 *ermB*-carrying isolates were resistant to erythromycin and clindamycin. The two isolates carrying *linB* were resistant to clindamycin and susceptible to erythromycin. All 24 isolates carrying *mefA*/*E* were resistant to erythromycin and susceptible to clindamycin. The three isolates carrying *ermA* were resistant to clindamycin and erythromycin. The remaining 11 isolates carrying *ermA* were resistant to erythromycin, susceptible to clindamycin, and showed erythromycin-induced clindamycin resistance. The two isolates carrying *ermA* and *msrD* were resistant to erythromycin and clindamycin. The seven isolates carrying *mefA*/*E* and *msrD* were resistant to erythromycin and susceptible to clindamycin.

### Penicillin- and cephem-resistant isolates.

Seven isolates (2.6% [7/268]) were resistant to penicillin G; one isolate, four isolates, one isolate, and one isolate were of serotypes Ia, Ib, II, and III, respectively. Four isolates, five isolates, and one isolate were resistant to ampicillin, cefazolin, and cefotaxime, respectively (Table 4). Three isolates were resistant to penicillin G and cefazolin, two to penicillin G and ampicillin, one to penicillin G, ampicillin, and cefazolin, and one to penicillin G, ampicillin, cefazolin, and cefotaxime. Penicillin G-resistant isolates were not necessarily resistant to other penicillin- and cephem-based antibiotics. Additionally, the antibacterial resistance differed, depending on the strain. Not all strains were resistant to the same antimicrobial agents; therefore, specific resistant strains were probably not prevalent in the community.

**TABLE 4:** MIC and molecular characterization of penicillin-resistant GBS with STSS

Strain	MIC (μg/mL) of^*a*^:					Serotype	Outcome	Amino acid substitution(s)^*b*^			Subclass^*c*^
	Pen G	Amp	Cfz	Ctx	Cli			PB1A	PBP2B	PBP2X	
PR1	0.25	0.5	0.5	0.25	≥16	Ia	Live	T537P	T567I	I377V	New class
								F541V		G398A	
								N635-		Q412L	
								G636-		H438Y	

PR2	0.25	0.25	2	0.5	≥16	Ib	Live	A621V		G398A	IIc
										**V405A**	
										**Q557E**	

PR3	0.5	0.25	2	0.5	≥16	Ib	Dead	A547V		G398A	IIc
										**V405A**	
										**Q557E**	

PR4	0.25	0.5	1	0.25	0.12	Ib	Live		T567I	I377V	IIIa
										F395L	
										**V405A**	
										R433H	
										H438Y	
										T473M	
										V510I	
										G648A	

PR5	0.25	0.5	1	1	≥16	Ib	Live		T567I	G329V	IIIa
										K372E	
										G398A	
										**V405A**	
										G429D	

PR6	0.25	0.25	1	0.5	≥16	II	Dead		L269Q	G398A	IIIc
									T567I	**V405A**	
										**Q557E**	

PR7	0.25	0.5	0.5	0.25	0.12	III	Live	F541V	T567I	I377V	New class
								N635-		G398A	
								G636-		Q412L	
										H438Y	

a Pen G, penicillin G; Amp, ampicillin; Cfz, cefazolin; Ctx, cefotaxime; Cli, clindamycin. Each MIC value was consistently obtained across three independent assays.

b Substitutions strongly associated with penicillin-resistant GBS are shown in boldface.

c Subclasses were determined according to Kimura et al. (21).

Sequence analysis of *pbp* was performed for each of the seven penicillin G-resistant GBS isolates. The penicillin G-resistant GBS strains had several amino acid substitutions in the deduced amino acid sequence of the penicillin-binding protein PBP2X. In contrast, either or both PBP1A and PBP2B had amino acid substitutions. The V405A and Q557E amino acid substitutions commonly detected in PBP2X of penicillin-resistant strains were found in five of the seven strains (Table 4). According to Kimura et al. (21), PBPs are classified according to amino acid substitutions. Two strains belonged to subclasses IIc and IIIa, and one belonged to subclass IIIc. In addition, two strains belonged to a new class (Table 3).

Five of the seven penicillin-resistant GBS isolates (71.4%) were clindamycin resistant. Regarding outcomes for patients with penicillin-resistant GBS isolates, two of the seven patients (28.6%) died. Regarding outcomes in patients from whom penicillin G- and clindamycin-resistant GBS strains were isolated, two of the five patients (40.0%) died.

### Ciprofloxacin-nonsusceptible isolates.

Of the 268 isolates, 6 (2.2%) were intermediately resistant to ciprofloxacin, and 90 (33.6%) were resistant to ciprofloxacin. Ciprofloxacin-resistant isolates were serotypes Ia (four isolates), Ib (71 isolates), II (one isolate), III (nine isolates), and V (four isolates), and one isolate was untypeable. Of the serotype Ib isolates, 92.2% (71/77) were ciprofloxacin resistant.

## DISCUSSION

We investigated 268 STSS cases caused by GBS in nonpregnant adults. Serotype Ib was the most prevalent, followed by serotypes V, III, and Ia, which were identified in over 80% of STSS isolates. Of the 268 isolates, 48 clindamycin-resistant (17.9%) and 7 penicillin G-resistant (2.6%) strains were isolated between 2014 and 2021. Clindamycin-resistant isolates had *linB*, *ermA* (including two isolates with *ermA* and *msrD*), or *ermB* genes. Penicillin G-resistant isolates contained amino acid substitutions in PBP2X and PBP1A or PBP2B. There have been studies on invasive GBS infections; however, this is the first to study strains isolated from over 100 STSS cases.

According to active, population-based surveillance for invasive GBS disease via the Active Bacterial Core surveillance network in the United States, the median age and case fatality rate of invasive GBS infection were 64 years and 5.6%, respectively. On the other hand, those for STSS were 73 years and 24.6%, respectively. The median age in patients with STSS was higher than that in invasive GBS infections. This may mean that older patients are more likely to progress to STSS. This may have resulted in increased severity and fatality rates.

In Japan, serotypes of isolates obtained from infants with invasive infections have been reported (22). The rates of serotypes Ib (*P* < 0.001) and V (*P* < 0.001) in strains isolated from STSS cases (this study) were significantly higher than those in strains isolated from infants with invasive infections (22). In contrast, serotypes III (*P* < 0.001) and Ia (*P* = 0.0192) were isolated more often in infants with invasive infections than in STSS isolates in this study (22). These data suggest that the serotype distributions of isolates caused by STSS and invasive infections in infants differ.

Various GBS vaccine formulations have been tested in clinical trials but are not currently approved (23). Based on this study’s findings, the trivalent and hexavalent conjugate vaccines have efficacies of 61.2% (164/268) and 91.4% (245/268), respectively, for preventing STSS from GBS. The coverage of the hexavalent conjugate vaccine was considerably higher than that of the trivalent vaccine because the hexavalent conjugate vaccine contained the second most frequently isolated serotype V polysaccharide (Fig. 1). Therefore, vaccination of nonpregnant adults with the hexavalent conjugate vaccine could reduce STSS cases due to GBS by over 90%.

The serotype distribution of GBS causing invasive infections in nonpregnant adults has been reported in various countries (24). Serotype V was the most prevalent serotype globally (43.5%) and in North America (46.7%). On the other hand, according to active, population-based surveillance for invasive GBS disease via the Active Bacterial Core surveillance network in the United States in 2008 to 2016, serotype Ia was the most prevalent serotype (5). Furthermore, serotype Ia was the second most prevalent serotype, followed by serotype III in Europe (25.0%) and Asia (29.5%). In Japan, the most prevalent serotype was Ib (28.5%) in isolates from patients with STSS (Fig. 1). Additionally, before 2013, serotype Ib was the most prevalent in STSS (11) and invasive infections (7). These findings suggest that GBS serotype distribution may differ geographically among nonpregnant adults, particularly in Japan.

In 15 of 19 studies, strains isolated from invasive GBS infection were susceptible to penicillin. Four studies reported some resistance to penicillin in Thailand (2%), Latin America (1.4%), the United States (0.5%), and Taiwan (2%) (5, 24). In our study, seven isolates (2.6%) were resistant to penicillin, and the rates of penicillin-resistant isolates were similar to those of these studies. In Japan, the rate of penicillin-resistant strains isolated from invasive GBS infections in 2010 to 2013 was 0% (7). Therefore, paying attention to the emergence of penicillin-resistant strains in recent years in Japan is necessary.

This study's proportion of erythromycin-resistant isolates was high (30.2%) (Table 2). This high resistance rate is recognized in Japan and worldwide (24) and may pose a serious challenge in clinically managing patients allergic to penicillin. A recent study in Portugal reported an increase in macrolide resistance in GBS despite its decreased consumption, suggesting that the expansion of specific clones is the main factor for this variation (25). Thus, constantly monitoring changes in the GBS epidemic and implementing GBS vaccination covering a wide range of serotypes may help to reduce antimicrobial resistance.

We collected 268 GBS isolates from patients with STSS. Five isolates were resistant to penicillin and clindamycin (Table 4). Two of the five patients (40.0%) died. This is the first report of mortality in patients with STSS caused by penicillin- and clindamycin-resistant GBS strains.

Notably, our study had some limitations. Patient data on antibiotic history and clinical characteristics were unavailable. Therefore, we focused on outcomes and analysis of *in vitro* data. Further investigation of the outcome and timing of antibiotic treatment in penicillin- and clindamycin-resistant strains is warranted. This study has interpretive limitations due to a lack of sequence type or clonal complex analysis.

Herein, we presented GBS isolated from over 100 patients with STSS in Japan. The distribution of serotypes differed from that worldwide and is characteristic of Japan. Penicillin-resistant bacteria were also isolated from GBS from patients with STSS; many isolates were also resistant to clindamycin. Therefore, introduction of the GBS vaccine, particularly the hexavalent conjugate, is vital to preventing infection and reducing the number of patients with STSS. Ongoing monitoring using antimicrobial susceptibility testing and serotyping of GBS that caused STSS will help monitor resistance to penicillin and clindamycin, lethality impact, and circulating serotypes.

## MATERIALS AND METHODS

### Bacterial isolates.

STSS is classified as notifiable by the Infectious Disease Control law in Japan. Cases of STSS due to GBS are reported annually: 2014, 31; 2015, 36; 2016, 59; 2017, 80; 2018, 87; 2019, 123; 2020, 121; 2021, 127. STSS-causing GBS isolates were obtained from pathogen collections of the National Institute of Infectious Diseases and prefectural Public Health Institutes (PHIs) from all over Japan. Data on streptococcal infections and clinical isolates were sent to PHIs from cooperating hospitals. We collected data from seven reference center branch offices in the PHIs of Fukushima, Tokyo, Kanagawa, Toyama, Osaka, Yamaguchi, and Oita. For this study, adults were defined as individuals over 18 years old. The diagnostic criteria for STSS were established by the Centers for Disease Control and Prevention in 1993 (8). They included identification of GBS from a normally sterile site, septic shock, and multiorgan failure. All GBS isolates obtained were cultured from sterile body sites of patients with STSS. Isolates were part of standard patient care. This study complied with the guidelines of the Declaration of Helsinki and was approved by the institutional individual ethics committees for the use of human participants (National Institute of Infectious Diseases Ethic Review Board for Human Subjects approval no. 19). The study was considered public health surveillance as defined in Article 15 of the Act on the Prevention of Infectious Diseases and Medical Care for Patients with Infectious Diseases (1999). Thus, informed consent was not required. An API 20 Strep kit was used to identify the strains (bioMérieux, Marcy l'Étoile, France). The Lancefield grouping was determined using the Prolex streptococcal grouping latex kit (Iwaki & Co., Japan).

### Capsular serotyping.

Capsular serotyping of each isolate was performed using the latex agglutination method with specific antisera against Ia to IX capsular polysaccharide antigens (Statens Serum Institute, SSI Diagnostica, Denmark).

### Antimicrobial susceptibility tests.

The antimicrobial susceptibility of the isolates was assessed using the broth microdilution method, as recommended by the Clinical and Laboratory Standards Institute (CLSI) (26). The resistance breakpoints for each drug, excluding those for cefazolin and ciprofloxacin, were recommended by the CLSI: for penicillin G, ≥0.25 μg/mL; ampicillin, ≥0.5 μg/mL; cefotaxime, ≥1 μg/mL; erythromycin, ≥1 μg/mL; clindamycin, ≥1 μg/mL; meropenem, ≥1 μg/mL; vancomycin, ≥2 μg/mL; daptomycin, ≥2 μg/mL; and linezolid, ≥4 μg/mL (26). An arbitrary resistance breakpoint of ≥1 μg/mL was used for cefazolin, and ≥8 μg/mL was used for ciprofloxacin; however, resistance breakpoints have not been established by the CLSI for beta-hemolytic *Streptococcus*.

### Detection of erythromycin resistance genes.

The genes responsible for erythromycin resistance, *ermA*, *ermB*, *mefA*, and *msrD*, were detected via PCR using previously published primer sequences (see Table S1 in the supplemental material) (27–29). Briefly, the reaction mixture (25 μL) contained 1 μL DNA template, 1 μL 10 mM each primer, 0.2 μL 5 U/μL AmpliTaq Gold DNA polymerase, 2.5 μL 10 mM deoxynucleoside triphosphates, 2.5 μL 10×AmpliTaq Gold buffer (2.5 mM MgCl_2_ Plus; Applied Biosystems, Waltham, MA, USA), and 17.3 μL water. The PCR conditions were 95°C for 20 s, 48°C for 25 s, and 72°C for 1 min for 30 cycles, followed by an initial denaturation of 95°C for 10 min and a final extension step of 72°C for 1 min.

### Analysis of *pbp*.

Five genes encoding high-molecular-weight penicillin-binding proteins (PBP1a, PBP1b, PBP2a, PBP2b, and PBP2x) were amplified and sequenced in STSS isolates as described previously (30). The sequencing products were compared with the S. agalactiae A909 NCBI reference strain.

### Statistical analysis.

Statistical analyses were performed using EkuseruToukei v.4.02 (Social Survey Research Information Co., Ltd., Tokyo, Japan). Data were compared using Fisher’s exact test. Differences were considered significant at *P* values of <0.05.

### Data availability.

The nucleotide sequences of the *pbp* genes from all penicillin G-resistant GBS strains tested in this study were deposited in EMBL/GenBank through DDBJ under accession no. LC731361 to LC731376.
