# Supplementary material for: Serotype Distribution and Antimicrobial Resistance of Streptococcus agalactiae Isolates in Nonpregnant Adults with Streptococcal Toxic Shock Syndrome in Japan in 2014 to 2021
Source: Microbiol Spectr. 2023 Feb 14;11(2):e04987-22. doi: 10.1128/spectrum.04987-22 (PMC10100893; doi:10.1128/spectrum.04987-22)
Supplement: Supplemental file 1 — Supplemental material. Download spectrum.04987-22-s0001.pdf, PDF file, 0.02 MB [file spectrum.04987-22-s0001.pdf]

Supplementary Table 1. Primers used for detecting erythromycin resistance genes

**Gene**	**Primer name**	**Primer sequence (5′-3′)**
mefA/E	mefA1	AGT ATC ATT AAT CAC TAG TGC
	mefA2	TTC TTC TGG TAC TAA AAG TGG
ermA	ermTR1	GAA GTT TAG CTT TCC TAA
	ermTR2	GCT TCA GCA CCT GTC TTA ATT GAT
ermB	ermB1	GAA AAG GTA CTC AAC CAA ATA
	ermB2	AGT AAC GGT ACT TAA ATT GTT TAC
msrD	msrD-F	TTGGACGAAGTAACTCTG
	msrD-R	GCTTGGCTCTTACGTTC
